# Stathmin Potentiates Vinflunine and Inhibits Paclitaxel Activity

**DOI:** 10.1371/journal.pone.0128704

**Published:** 2015-06-01

**Authors:** Soazig Malesinski, Philipp O. Tsvetkov, Anna Kruczynski, Vincent Peyrot, François Devred

**Affiliations:** 1 Aix-Marseille Université, Inserm, CRO2 UMR_S 911, Faculté de Pharmacie, Marseille, France; 2 Institute of General Pathology and Pathophysiology, RAMS, Moscow, Russian Federation; 3 Centre de Recherche en Oncologie Expérimentale, Centre de Recherche et Développement Pierre Fabre, Toulouse, France; Stanford University School of Medicine, UNITED STATES

## Abstract

Cell biology and crystallographic studies have suggested a functional link between stathmin and microtubule targeting agents (MTAs). In a previous study we showed that stathmin increases vinblastine (VLB) binding to tubulin, and that conversely VLB increases stathmin binding to tubulin. This constituted the first biochemical evidence of the direct relationship between stathmin and an antimitotic drug, and revealed a new mechanism of action for VLB. The question remained if the observed interaction was specific for this drug or represented a general phenomenon for all MTAs. In the present study we investigated the binding of recombinant stathmin to purified tubulin in the presence of paclitaxel or another *Vinca* alkaloid, vinflunine, using Isothermal Titration Calorimetry (ITC). These experiments revealed that stathmin binding to tubulin is increased in the presence of vinflunine, whereas no signal is observed in the presence of paclitaxel. Further investigation using turbidity and co-sedimentation showed that stathmin inhibited paclitaxel microtubule-stabilizing activity. Taken together with the previous study using vinblastine, our results suggest that stathmin can be seen as a modulator of MTA activity and binding to tubulin, providing molecular explanation for multiple previous cellular and in vivo studies showing that stathmin expression level affects MTAs efficiency.

## Introduction

Microtubules (MTs) are major components of the cytoskeleton, whose dynamics and functions in cell division and cell motility are targeted in cancer therapy. Microtubule targeting agents (MTAs), divided into microtubule-stabilizing (e.g., taxanes) and microtubule-destabilizing agents (e.g., *Vinca* alkaloids), constitute a major anti-cancer drug family used in treatment of a wide variety of human cancers [1]. Ever since their discovery, there is a continuous effort to understand the molecular mechanisms of MTAs and to develop new microtubule inhibitors to improve anticancer therapies and circumvent drug resistance [1–3]. One of the challenges is to understand why some drugs are efficient in certain clinical cases and not in others. Microtubule associated proteins (MAPs) are among the markers that are suspected to modulate the efficacy of a MTA and thus predict the outcome of a treatment, since they target tubulin and have stabilizing or destabilizing activity on microtubules.

Over the past ten years, many studies suggested a role for stathmin in cancer [4–7] and in the modulation of MTAs effect [8–11]. Here, we investigated the links between stathmin and MTAs at the molecular level. Stathmin is known to promote microtubule depolymerization by sequestrating free tubulin in a T2S complex and by binding and curving protofilaments at microtubule ends [12,13]. Recently, we presented the first molecular evidence of a direct functional interplay between a MTA (vinblastine) and stathmin *in vitro* [14]. In the present study, we investigated the functional interaction between stathmin and two other MTAs to see if the interplay between MTAs and stathmin was a general molecular phenomenon. Using isothermal titration calorimetry (ITC) which was successfully used to study stathmin or tau binding to tubulin previously [14–17], we demonstrated that vinflunine [18] and paclitaxel both affect thermodynamics of stathmin binding to tubulin. Additional turbidimetry and cosedimentation assay further showed that in the presence of stathmin, paclitaxel microtubule polymerizing activity is greatly impaired. Based on these results, we proposed the molecular mechanism by which stathmin can modulate the actions both of destabilizing MTAs such as *Vinca* alkaloids and of stabilizing agents such as paclitaxel.

## Material and Methods

### Protein purifications

Tubulin was purified from lamb brains, stored and dosed as described previously [19]. Lamb brains were purchased from Alazard & Roux slaughterhouse (Tarascon, France). Human recombinant stathmin was purified from *E*. *coli*, stored, equilibrated and dosed as described previously [14].

### Isothermal Titration Calorimetry (ITC)

Binding of stathmin to tubulin was analyzed by ITC using MicroCal VP-ITC as described previously [15,20]. Experiments were performed at 37°C rather than at 10°C and in 20 mM NaPi buffer in the presence of 4 mM MgCl_2_, 0.1 mM GTP, pH 6.5 in order for paclitaxel to be active. Tubulin concentrations in the calorimetric cell ranged from 5 to 20 μM, whereas stathmin concentrations in the syringe varied from 50 to 200 μM. When the experiment was conducted in the presence of MTA, it was added both in the calorimetric cell (with tubulin) and in the syringe (with stathmin). Tubulin was titrated by repeated injections of 10 μL aliquots of stathmin. Each resulting titration peak was integrated and plotted as a function of the stathmin/tubulin molar ratio. The baseline was measured by injecting stathmin into the protein-free buffer solution. Data were analyzed using the FIT software developed in CRO2 (Aix Marseille University, France) and were fitted with a “one set of sites” model via a non-linear least squares minimization method and led to the determination of apparent affinity constants (K_app_) and enthalpy changes (ΔH). The affinity constants determined are an average of at least three different experiments.

### Turbidimetry

Microtubules polymerization was monitored by turbidimetry on a Perkin-Elmer spectrophotometer Lambda 800 at 350nm. Purified tubulin (10μM) was equilibrated in PM buffer (20mM NaPi, 4mM MgCl_2_, pH 6.5, 0.1mM GTP) and incubated at 37°C. Polymerization was initiated by adding 10μM of paclitaxel. Purified stathmin (0–15μM) was incubated 10 minutes before or 10 minutes after polymerization induction.

### Co-sedimentation assay

Microtubules polymerized either in PM buffer or in PEM buffer (80mM PIPES pH 6.8, 2 mM Mg Cl_2_, 1mM EGTA, 0.1mM GTP) were separated from tubulin and stathmin oligomers by sedimentation at 50000 rpm, 20 minutes at 37°C in an TL-100 Beckman ultracentrifuge with a TLA 100.2 rotor. Pellets were resuspended in cold PM or PEM buffer. Supernatants and pellets were analyzed by 15% SDS PAGE gels. Proteins were stained with Coomassie Brilliant Blue and band intensity quantification by Image J was performed on gels where the loading of each sample was adjusted to obtain comparable and non-saturated spots. Results presented are average of at least 3 experiments for each condition. Error bars correspond to standard deviation scores.

## Results

### Thermodynamic parameters of stathmin binding to tubulin are modified in the presence of microtubule targeting agents

**Fig 1:**
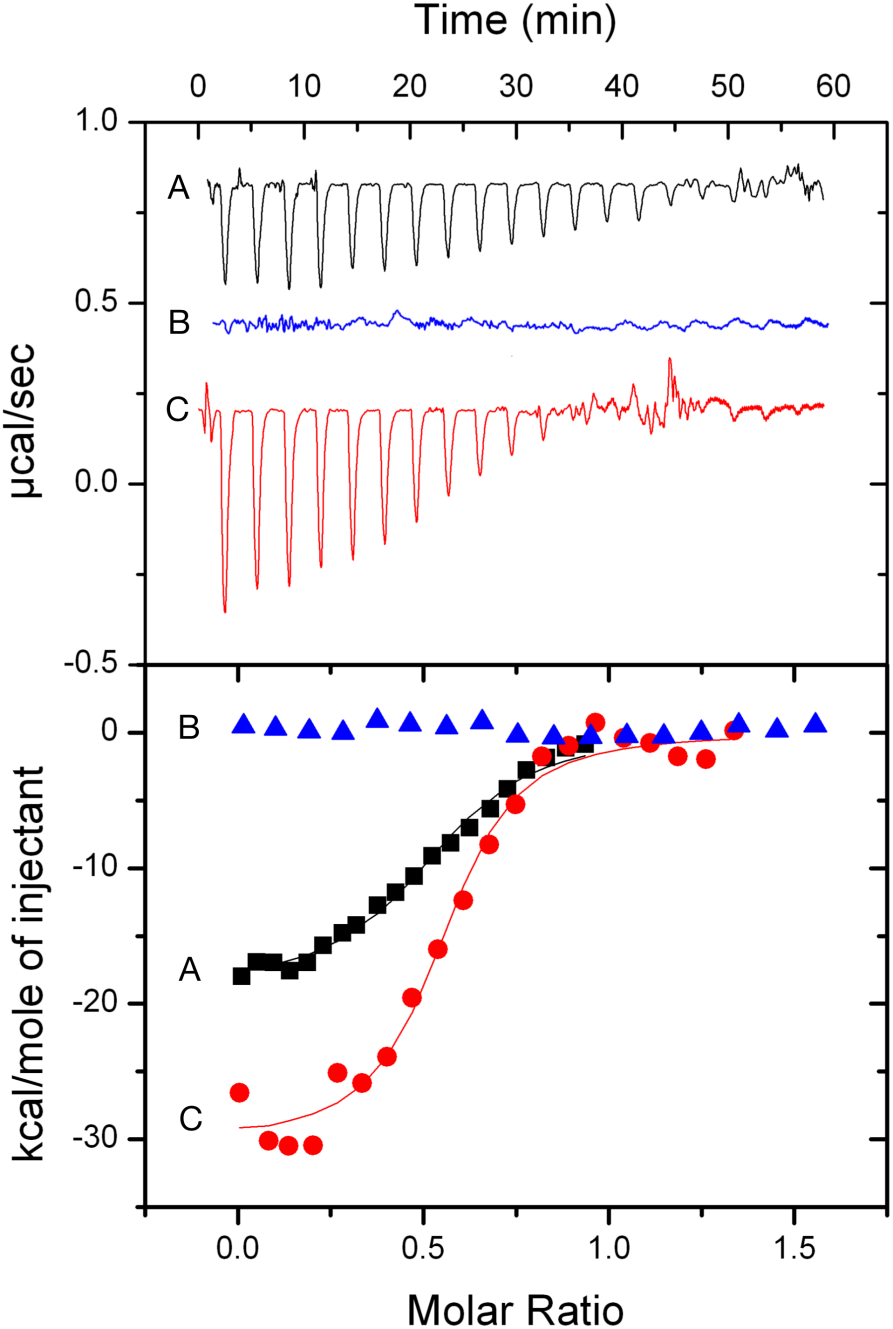
Thermodynamic analysis of stathmin-tubulin interaction. Vinflunine increases stathmin binding to tubulin whereas paclitaxel does not. Typical curves of: **A**: raw data obtained for 25–30 injections, each of 10 μL of stathmin (80 μM) into the sample cell containing tubulin (10 μM) in the presence or the absence of MTAs. The three titration curves (which were translated upward for clarity purposes) were obtained during one set of experiments; curve A corresponds to stathmin binding to tubulin in the absence of MTA, curve B corresponds to stathmin binding to tubulin in the presence of vinflunine (10μM), whereas curve C corresponds to stathmin binding to tubulin in the presence of paclitaxel (10μM). **B**: integrated heats of reaction (symbols) in the absence of MTAs (squares), in the presence of vinflunine (circles) and in the presence of paclitaxel (triangles) with the best fit to the data (lines). The measurements were carried out at 37°C in PM buffer (20 mM NaPi, GTP 0.1 mM MgCl_2_ 4mM pH 6.5).

Having previously shown by isothermal titration calorimetry (ITC) that stathmin binding to tubulin was affected by vinblastine [14], we investigated the binding of recombinant stathmin to purified tubulin in the presence of paclitaxel or another *Vinca* alcaloid, vinflunine, using the same approach. Fig 1 (upper panel) shows the typical exothermic profiles obtained for tubulin titration by stathmin at 37°C either without MTAs (curve A), in the presence of vinflunine (curve B) or in the presence of paclitaxel (curve C). Fig 1 (lower panel) shows binding isotherms, *i*.*e*. the integrated heats of reaction as a function of the stathmin/tubulin molar ratio in these three conditions. By fitting the binding isotherm without MTAs (curve A), we determined a ΔH value of -7.5 kcal/mole of stathmin, a ΔS value of -30.9 cal/mol/deg and a tubulin–stathmin binding constant of 2.7 ± 0.4 × 10^6^ M^-1^. Contrary to thermodynamic parameters obtained for this interaction at 10°C, for which the binding is endothermic (ΔH >0; enthalpy unfavorable), and entropy driven (ΔS >0), at 37°C this interaction is exothermic (ΔH <0; enthalpy driven) and entropy unfavorable [14,21]. This difference could be explained by temperature dependent changes in dimerization interface between two successive tubulins. Indeed, tubulin polymer formation is known to be dependent on temperature. For example, a same preparation of tubulin will induce rings at 10°C and microtubules at 37°C [19]. In the presence of vinflunine (curve B), the variation of negative enthalpy (ΔH) of stathmin-tubulin interaction was increased to -30kcal/mole of stathmin, the ΔS to -65.7 cal/mol/deg and a tubulin–stathmin binding constant value of 7.8 ± 2.0 × 10^6^ M^-1^ was found. Thus, in the presence of vinflunine, stathmin affinity for tubulin increased 3-fold. This process was even more entropy unfavorable than in absence of MTA, indicating that vinflunine favors stathmin binding to tubulin and that the complex formed should be more rigid. In contrast, in the presence of paclitaxel which induces the formation of MTs, the variation of enthalpy of stathmin-tubulin interaction is diminished, indicating that stathmin binding to paclitaxel-stabilized MTs at 37°C is driven by favorable entropy changes. Even though at this point the absence of detectable signal in the presence of paclitaxel did not enable us to distinguish between a simple change in the environment leading to a variation of ΔH and a possible absence of binding, these results showed that both depolymerizing and stabilizing MTAs directly affect stathmin activity by modulating its binding thermodynamics to tubulin.

### Stathmin inhibits paclitaxel microtubule polymerizing activity

**Fig 2:**
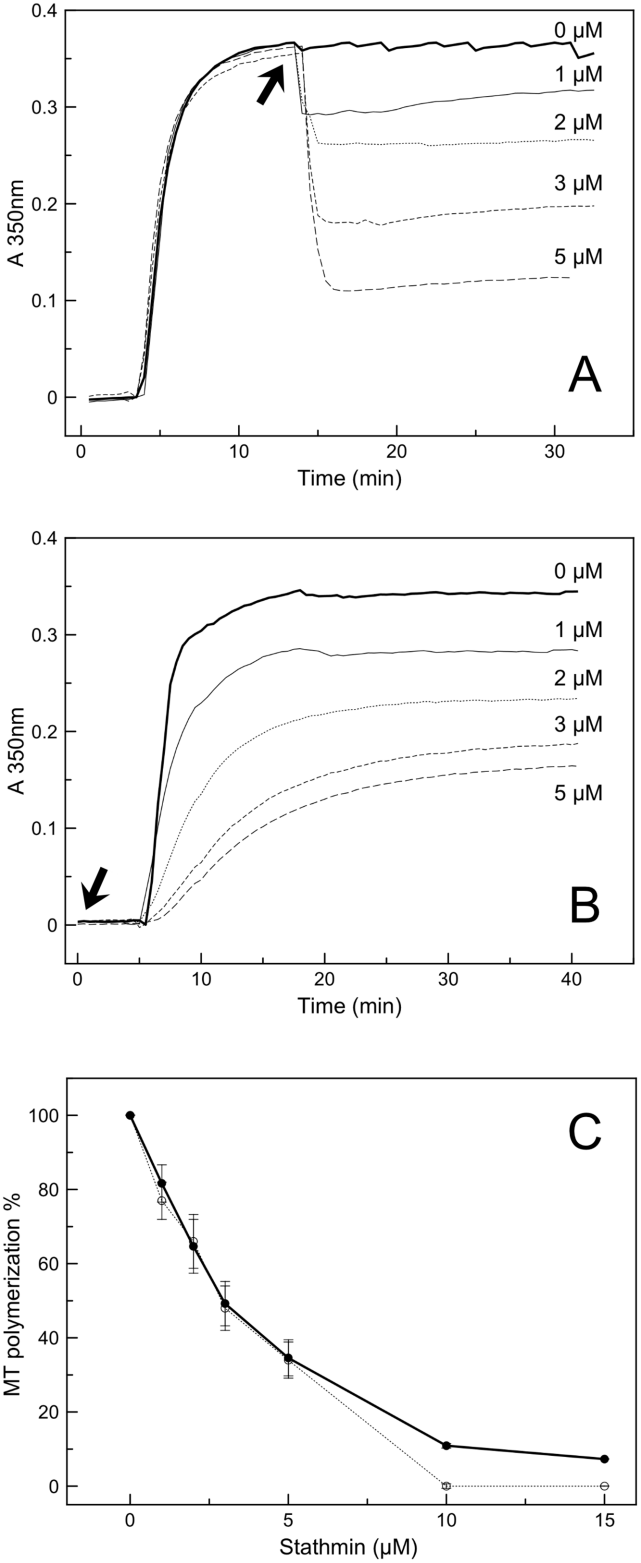
Effect of stathmin on *in vitro* microtubules polymerization in PM buffer followed by turbidity. Various concentrations of stathmin ranging from 0 to 15μM were added at the arrow to 10μM tubulin after (panel A) or before (panel B) addition of 10μM paclitaxel. Panel A and B are representative turbidimetric curves of samples treated with 0, 1, 2, 3, or 5μM stathmin. Panel C represents the average of at least three experiment for each stathmin concentration (0, 1, 2, 3, 5, 10 and 15μM). Solid line corresponds to addition of stathmin after paclitaxel (panel A conditions) and dotted line corresponds to addition of stathmin before paclitaxel (panel B conditions) showing that the effect of stathmin on polymerization is similar whether stathmin is added before or after paclitaxel.

**Fig 3:**
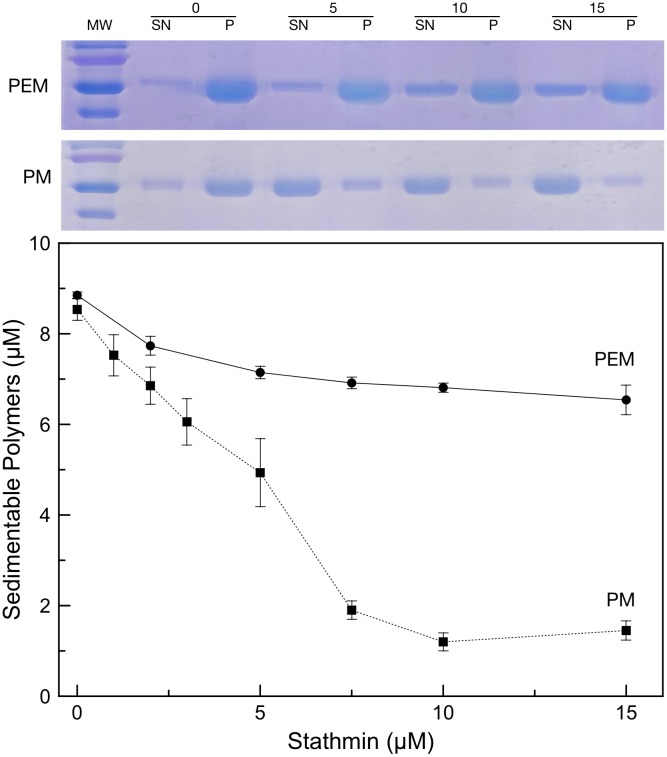
Effect of the buffer on stathmin depolymerizing activity. Samples containing various concentrations of stahtmin (0–15μM) added to paclitaxel stabilized microtubules (10μM tubulin) were sedimented and the amount of tubulin in the supernatants (SN) and pellets (P) was analyzed by SDS PAGE. The two representative gels displayed above the graph correspond to samples in the presence of 0, 5, 10 or 15μM stathmin, in PEM buffer (upper gel) or PM buffer (lower gel). Quantification of sedimentable tubulin polymers assembled in PM buffer (black squares) vs. PEM buffer (black circle) corresponds to averages of 0, 1, 2, 3, 5, 7.5, 10 and 15 μM stathmin in PM buffer and 0, 2, 5, 7.5, 10 and 15 μM stathmin in PEM buffer.

To find out if the absence of detectable heat exchanged measure corresponded to an absence of binding of stathmin to tubulin in the presence of paclitaxel, we measured the depolymerizing activity of stathmin on paclitaxel-stabilized microtubules. Using turbidimetry, we monitored the formation of microtubules in a minimal Phosphate/Mg^2+^ buffer (PM buffer, see Material and Methods). The use of such buffer was chosen to avoid potential side effects of known pro-polymerizing factors such as glycerol or PIPES. In our conditions, addition of paclitaxel was necessary and sufficient to induce microtubule formation (Fig 2). Turbidimerty experiment revealed that when stathmin is added on paclitaxel-induced microtubules it triggers a fast depolymerization, in a concentration dependent manner (Fig 2A). Likewise when stathmin is added prior to addition of paclitaxel we also observed a concentration dependent inhibition of microtubule polymerization (Fig 2B). It should be noted that the same level of polymerization are reached whether stathmin is added before or after paclitaxel (Fig 2C). Inhibition of polymerization by stathmin shown by turbidimetry measurements in PM buffer (S1 Fig) was then confirmed by co-sedimentation assay coupled to quantitative analysis of pellets containing MTs by SDS PAGE (Fig 3, second gel and black squares). Indeed, quantification of the amount of polymerized tubulin showed that in the presence of stathmin, the amount of MTs decreases, indicating that paclitaxel is losing its pro-polymerizing activity. Interestingly, when the same experiment was conducted in presence of additional polymerizing promoters, such as PIPES, no significant decrease in turbidity or microtubule mass by co-sedimentation were observed (S1 Fig and Fig 3, first gel and black circles respectively), indicating that stathmin was no longer able to depolymerize MT formed in this condition. This is in agreement with similar experiments also conducted in PIPES, in which stathmin seems to lose its ability to depolymerize paclitaxel-stabilized microtubules [22].

## Discussion

While studying interplay between stathmin and microtubule targeting agents, we have previously showed that vinblastine induced a strong increase of stathmin binding to tubulin and vice versa [14], providing an explanation for the increased sensitivity to *Vinca* alkaloids in cells over-expressing stathmin [8]. In the current study, we showed *in vitro* that stathmin binding to tubulin was also increased in the presence of vinflunine, enhancing the depolymerizing effect of this MTA. However, contrary to vinblastine, which induced a 50-fold increase in stathmin affinity for tubulin, vinflunine enhanced affinity 3-fold. This can be explained by the stronger affinity of vinblastine for tubulin compared to vinflunine [23,24]. Since members of this family of drugs share the same binding site [25], it is likely that all *Vinca* alkaloid molecules will similarly enhance tubulin-stathmin association by inducing the curving of consecutive tubulin dimers. Indeed, studies have shown that stathmin overexpression increases the sensitivity to other *Vinca* alkaloids, such as vindesine and vincristine in human lung carcinoma cells [26]. It is therefore possible that this mechanism could contribute to *Vinca* alkaloids selectivity for tumor cells, in which stathmin is over-expressed, compared to normal cells

In order to test whether stathmin would also affect the activity of MTAs from another class, we examined paclitaxel activity on tubulin polymerization into microtubules and found that it was inhibited by stathmin. It is important to note that our experiments were performed in phosphate buffer (PM buffer—see material and methods), which was different from previously published studies. Indeed, as shown by others [22] and confirmed by us in this study, stathmin does not inhibit paclitaxel-induced polymerization of MTs in PIPES buffer. The apparent discrepancy between results obtained in phosphate and PIPES buffer is not surprising, since it is known that PIPES, just like glycerol or sucrose, and unlike phosphate buffer, enhances tubulin polymerization into microtubule [27]. Thus, in PIPES conditions, stathmin inhibitory effect on paclitaxel activity is masked. In order to be able to attribute the observed polymerizing or depolymerizing effects to the molecules studied (here stathmin, vinflunine, or paclitaxel) it is necessary to use a minimal buffer deprived of any known stabilizing or destabilizing cofactors. Our results demonstrate for the first time that in the absence of additional polymerization promoter, intrinsic paclitaxel polymerizing activity is inhibited in the presence of stathmin. Our findings are consistent with recent clinical studies showing that high stathmin level are correlated with poor response or resistance to paclitaxel containing chemotherapy in endometrial or breast cancer [28–30] or reversely that functional knockdown of stathmin using siRNA results in increased sensitivity to paclitaxel [9,31].

**Fig 4:**
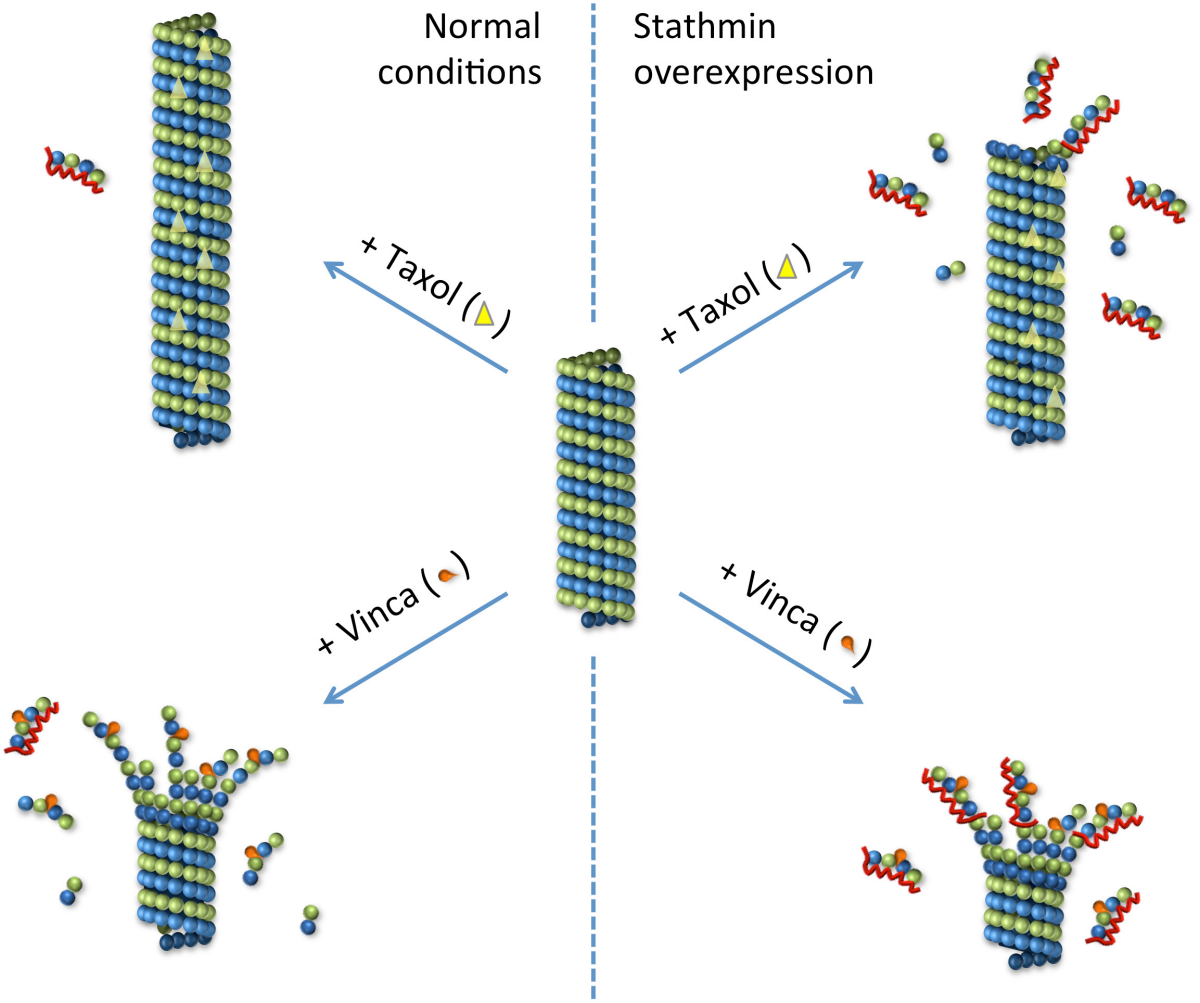
Model of the molecular mechanism of stathmin interplay with *Vinca* alkaloids and taxanes. Left side of the cartoon depicts a situation in which the level of expression is normal, *i*.*e*. not higher in the tumor that in the surrounding cells: stabilizing effect of paclitaxel and destabilizing effect of *Vinca* alkaloids, while right side depicts a situation where stathmin is overexpressed: loss of paclitaxel stabilizing effect and increase of *Vinca* alkaloids destabilizing effect.

Taken together, our findings provide a molecular mechanism by which stathmin can modulate MTAs effects on the equilibrium between tubulin and microtubules, as summarized in Fig 4.

While in tumors with normal stathmin levels paclitaxel has its expected stabilizing effect on microtubules and *Vinca* alkaloids their destabilizing effect, in stathmin-overexpressing tumors *Vinca* alkaloids effect is amplified and paclitaxel effect is impaired. This double mechanism is of great biological significance in clinics to target tumors that overexpress active stathmin compared to the normal surrounding tissues. Moreover, since activity of stathmin is influenced by phosphorylation [32–34], it would be very interesting to see if levels of stathmin as well as stathmin phosphorylation status differ between tumors responding and non-responding to specific MTAs.

Stathmin expression level is expected to be affected by mutations located in the promoter region as well as in the regulatory elements affecting transcription and splicing of this gene. In addition, mutations introducing a premature stop codon will also lead to decrease in full-length active stathmin production. These mutations could be acquired during tumor development (somatic mutations) or could represent germ-line polymorphisms present in a given patient. In the era of personal medicine, testing for stathmin mutations and expression level is likely to be a good marker to predict tumors sensitivity and response to pharmacological agents belonging to MTA family.

## Supporting Information

S1 Fig Effect of the buffer on stathmin depolymerizing activity monitored by turbidimetry. A. Turbidimetry signal of paclitaxel-stabilized microtubules (10μM tubulin) upon addition of various concentrations of stathmin (0–15μM). B. Turbidimetry plateau (reflecting the amount of microtubules) as a function of stathmin concentration in PM buffer (black circles) vs. PEM buffer (white squares) in presence of 0, 1, 2, 3, 5, 7.5, 10 and 15 μM stathmin in PM buffer and 0, 2, 5, 7.5, 10 and 15 μM stathmin in PEM buffer.
(TIFF)
