# Supplementary figures and images for: Stathmin Potentiates Vinflunine and Inhibits Paclitaxel Activity
Source: PLoS One. 2015 Jun 1;10(6):e0128704. doi: 10.1371/journal.pone.0128704 (PMC4451147; doi:10.1371/journal.pone.0128704)

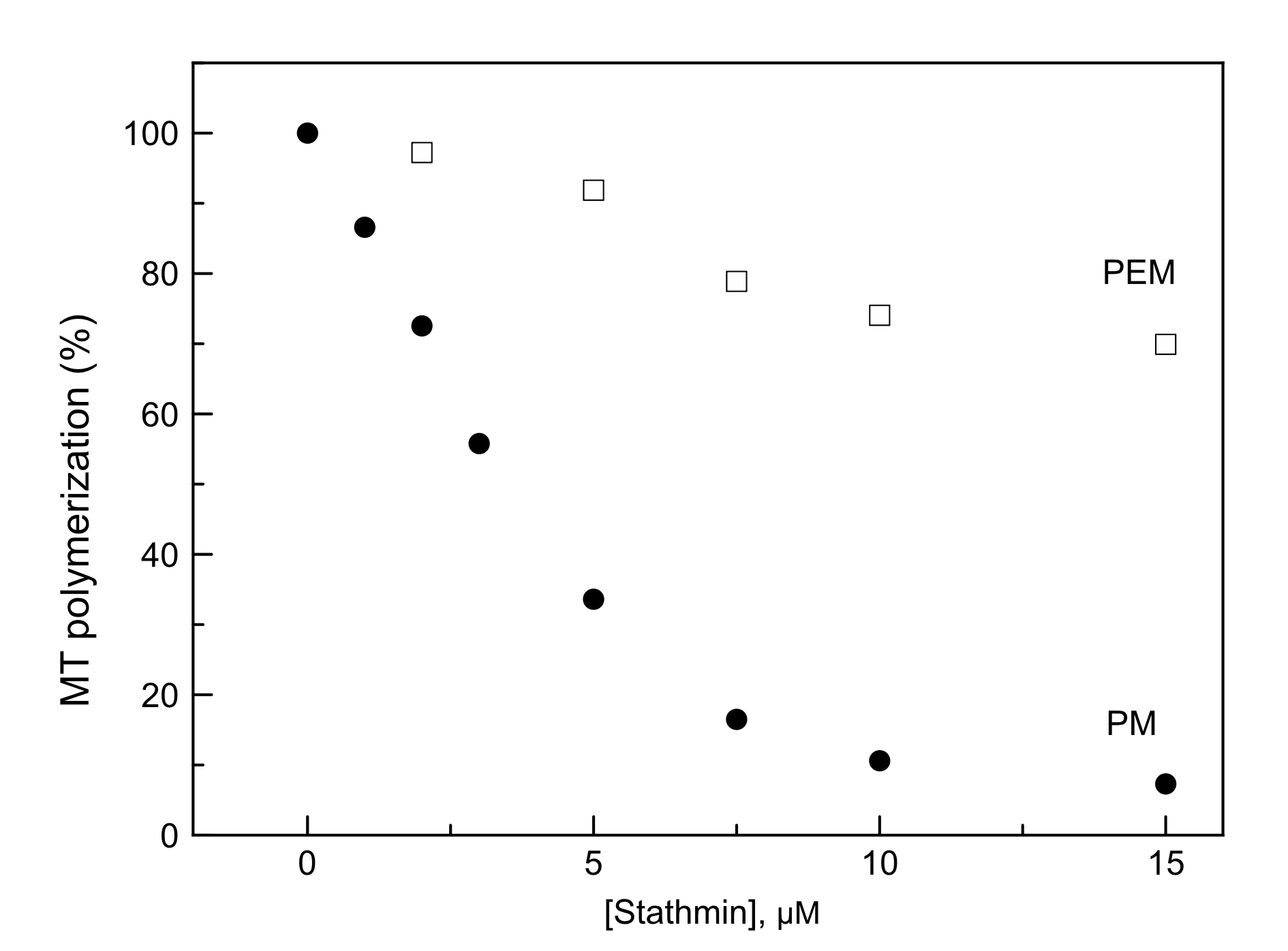

Supplement: S1 Fig — A. Turbidimetry signal of paclitaxel-stabilized microtubules (10μM tubulin) upon addition of various concentrations of stathmin (0–15μM). B. Turbidimetry plateau (reflecting the amount of microtubules) as a function of stathmin concentration in PM buffer (black circles) vs. PEM buffer (white squares) in presence of 0, 1, 2, 3, 5, 7.5, 10 and 15 μM stathmin in PM buffer and 0, 2, 5, 7.5, 10 and 15 μM stathmin in PEM buffer. (TIFF) [file pone.0128704.s001.tiff]
